# Polarity-Dependent Charge Transport and Resistance Degradation Enabled by Oxygen-Vacancy Gradients in BiFeO_3_ Films

**DOI:** 10.1021/acsami.6c00718

**Published:** 2026-06-23

**Authors:** Sengsavang Aphayvong, Abhyuday Verma, Kae Nakamura, Ali Habib Akhyari, Takeshi Yoshimura, Susan Trolier-McKinstry, Betul Akkopru-Akgun

**Affiliations:** † Department of Physics and Electronics Engineering, 639061 Osaka Metropolitan University, Sakai-Shi, Osaka 599-8531, Japan; ‡ Department of Materials Science and Engineering, 311285 The Pennsylvania State University, University Park, Pennsylvania 16802, United States

**Keywords:** Mn-doped BiFeO_3_, piezoelectric MEMS, oxygen vacancy gradient, conduction mechanism, electrical degradation, reliability

## Abstract

The long-term reliability of Mn-doped BiFeO_3_ thin films remains a key challenge for their implementation in piezoelectric microelectromechanical systems. In this work, the electronic transport mechanisms and DC resistance degradation of (100) Mn-doped BiFeO_3_ (BFMO) epitaxial films grown on (100) Si substrates by RF magnetron sputtering were systematically investigated. The leakage current characteristics exhibit a strong polarity dependence, originating from a nonuniform distribution of oxygen vacancies across the film thickness. Under field-up bias, where a high concentration of oxygen vacancies is present near the top interface, leakage is dominated by Poole–Frenkel emission from $V_{O}^{¨}$ related traps in the low-field regime and evolves into trap-controlled space-charge-limited conduction at higher fields, with transport controlled by ${\mathrm{Fe}}_{\mathrm{Fe}}^{\prime }$ sites. In contrast, under field-down bias, where the $V_{O}^{¨}$ concentration near the bottom interface is comparatively low, transport is Ohmic at low electric fields and transitions to Poole–Frenkel emission at higher fields, with conduction governed primarily by ${\mathrm{Fe}}_{\mathrm{Fe}}^{\prime }$ trap levels. The asymmetric $V_{O}^{\cdot \cdot }$ distribution also gives rise to polarity-dependent DC resistance degradation, governed by field-driven $V_{O}^{¨}$ drift and subsequent accumulation at the cathode interface. Since the $\left[V_{O}^{¨}\right]$ is initially higher near the top interface, $V_{O}^{¨}$ redistribution proceeds more rapidly, resulting in accelerated degradation kinetics and a reduced lifetime. The extracted $V_{O}^{¨}$ diffusion coefficient for BFMO (∼2.7 × 10^–14^ cm^2^ s^–1^) is approximately an order of magnitude higher than values reported for PZT films, consistent with the comparatively shorter lifetime observed under similar stress conditions. These findings highlight the pivotal role of defect engineering particularly in controlling oxygen vacancy concentration and migration for optimizing the reliability and performance of BiFeO_3_-based MEMS devices.

## Graphical abstract

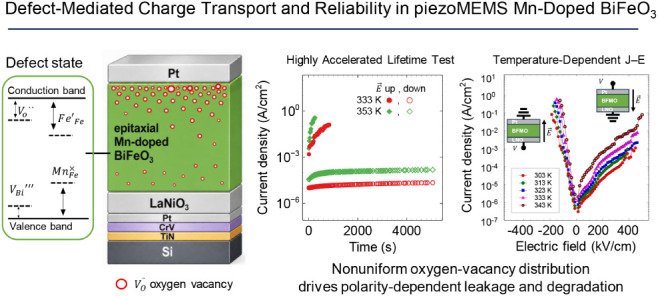

## 1 Introduction

Bismuth ferrite (BiFeO_3_, BFO) has attracted considerable attention in recent years as a multiferroic, owing to its high spontaneous polarization (∼100 μC/cm^2^), high Curie temperature (∼1100 K), piezoelectric responses, and potential applicability across nonvolatile memories, sensors, and piezoelectric microelectromechanical systems (piezoMEMS).^1–3^

Over the past decade, significant progress has been made in enhancing the electromechanical performance of BFO-based films through compositional doping and control of the microstructure and mechanical boundary conditions. For instance, Mn-doped BFO (BFMO) films have been reported with a high piezoelectric coefficient of *d*_31,*f*_ = 83 pm/V,^4^ while flexible films on a mica substrate were reported to show a remarkably high *d*_33_.^5^ These advances have increased interest in BFO-based films as lead-free piezoelectric materials for device-oriented applications, particularly in piezoMEMS.

Parallel advances in thin-film processing have enabled the integration of high-quality BFO films into MEMS-compatible platforms. Notably, (100)-oriented BFO films can be grown on (100) Si substrates using combinatorial radio frequency (RF) magnetron sputtering.^6,7^ BFO-based piezoMEMS devices have been reported for vibration energy harvesters, wireless power transfer systems, and acoustic sensors.^7–11^ These demonstrated applications are mainly passive, low-field, or self-powered operating modes, where the combination of a useful piezoelectric response and comparatively low dielectric permittivity is helpful. In these applications, BFO is advantageous because the piezoelectric sensing and energy harvesting figures of merit scale favorably with high piezoelectric response and low permittivity.

Moreover, epitaxially grown BFMO films on Si substrates have demonstrated efficient electromechanical energy conversion, benefiting from the strong piezoelectric response combined with comparatively low dielectric permittivity. In previous work using the same BFMO-on-Si platform, the BFMO film exhibited a dielectric constant of approximately 140, a dielectric loss tangent of approximately 1%, and an effective transverse piezoelectric coefficient *e*_31,_ *_f_* of −6.0C/m^2^. The optimized BFMO film was also integrated into a MEMS vibration-energy-harvester structure; an 800 nm-thick BFMO MEMS-pVEH showed *e*_31,*f*_ = −5.1 C/m^2^, *K* ^2^ = 0.5%, and *Q_m_* = 536, achieving a figure of merit up to 3.7 times higher than that reported for devices based on nondoped BFO-oriented films.^12^ A comparison of representative *e*_31,*f*_ of BFO-based films is provided in Table S1.^6,7,12–20^ These previously reported electromechanical results establish BFMO-on-Si as a MEMS-relevant piezoelectric film platform.

The next step toward broader implementation of BFO/BFMO piezoMEMS is to evaluate whether these films can operate reliably under electric field-driven conditions. Recently, BFO films have been proposed for piezoelectric micromachined ultrasonic transducers (pMUTs).^21^ This suggests the potential to extend BFO-based materials from predominantly passive sensing and energy harvesting applications toward actively driven ultrasonic transduction platforms. Unlike passive sensors or energy harvesters, actively driven piezoMEMS devices require repeated electric-field excitation and, in some cases, a DC bias component to generate mechanical motion or ultrasonic waves. Under these operating conditions, leakage current can reduce the actuation efficiency, increase local Joule heating, and accelerate electrical degradation. Thus, although BFO-based films are promising for piezoMEMS applications, their practical implementation in actively driven MEMS devices remains limited by leakage currents and field-induced degradation. Therefore, understanding leakage current mechanisms and field-induced degradation is essential for extending BFO/BFMO films from passive MEMS devices toward actively driven MEMS applications.

The high leakage current in BFO-based films is closely related to phase formation and defect chemistry.^22–24^ One of the challenges for BFO-based films is the formation of secondary phases such as Bi_2_O_3_, γ-Fe_2_O_3_, or Bi_25_FeO_39_ commonly reported during the fabrication process. These parasitic phases introduce unintended leakage paths in films, hence increasing leakage current and deteriorating the film’s overall electrical performance.^25–27^ Moreover, intrinsic point defects significantly affect the electrical reliability of BFO. Figure 1 illustrates the energy band diagram of BFO and shows a range of possible defect states important to the charge transport and conduction mechanism of the film.^28–35^ As can be seen, one critical contributor to leakage current is positively charged oxygen vacancies, for which the compensating electrons reside in shallow trap states and are easily excited into the conduction band. Oxygen vacancies lead to the local reduction of Fe^3+^ to Fe^2+^ for charge compensation, creating additional trap states. In addition, the mixed-valence Fe^2+^/Fe^3+^ also enables polaron hopping conduction, which contributes to a high leakage current of the film.^36^ Another intrinsic factor that causes high leakage current in BFO is the volatility of Bi^3+^ during fabrication.^37^ Bismuth deficiency not only leads to the formation of $V_{\mathrm{Bi}}^{\prime \prime \prime }$ but also enhances the concentration of oxygen vacancies to maintain local charge neutrality. These defect-related conduction pathways make electrical reliability under sustained or repeated electric fields a critical issue for BFO-based actuator-type MEMS devices.

**Figure 1:**
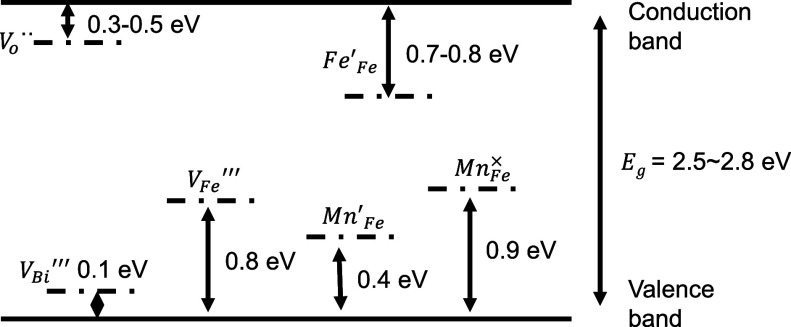
Schematic of the energy band structure of the BFMO film.

To address this, doping at either the A or B sites of the perovskite lattice with donor ions, such as Nb^5+^, has been shown to suppress the formation of oxygen vacancies that would otherwise compensate for the unbalanced charge produced due to Bi loss, thereby increasing the resistivity.^38^ Ba doping suppresses the formation of impurity phases (e.g., Bi_2_Fe_4_O_9_ and Bi_25_FeO_40_), which in turn enhances the electrical resistivity.^39^ In this work, Mn was selected because it directly targets the Fe-site defect chemistry associated with leakage in BFO. Since oxygen vacancies, Fe^2+^ formation, and Fe^2+^/Fe^3+^ hopping are major contributors to electronic conduction, Mn substitution is particularly relevant because prior studies have shown that Mn-doped BFO can reduce leakage current and improve ferroelectric properties by suppressing oxygen vacancy- and Fe^2+^-related conduction.^40–42^ Thus, Mn doping provides a targeted defect-engineering route for investigating charge transport and degradation in BFO-based films. In addition, Mn-doped BFO films on Si have already shown promising electromechanical performance in MEMS-relevant structures.^12^

To improve electrical resistivity and enable broader BFO/BFMO MEMS applications, it is essential to understand not only the conduction mechanisms but also their relationship to the defect chemistry and electrical degradation. While conduction mechanisms in BFO-based films have been widely investigated, a comprehensive understanding of their relationship with defect chemistry and electrical degradation under sustained DC bias in MEMS-compatible device configurations remains limited. This study focuses on clarifying the conduction mechanisms and DC resistance degradation of epitaxial BFMO films integrated on Si substrates,^12^ with particular emphasis on the relationship between charge transport behavior and defect chemistry. By addressing this material-level reliability bottleneck in a MEMS-compatible BFMO-on-Si, this work provides practical guidance for the future optimization of BFMO-based piezoelectric MEMS devices.

## 2 Experimental Methods

### 2.1 Sample Preparation

BFMO thin films were fabricated on 2-in.-diameter (100) Si wafers using RF magnetron sputtering.^12^ The bottom electrode was prepared using TiN as an epitaxial buffer layer, followed by a thin CrV adhesion layer, a Pt oxygen barrier layer, and a LaNiO_3_ (LNO) seed layer; the detailed procedure for bottom electrode preparation is described elsewhere.^43^ For BFMO deposition, sintered Bi_1.3_FeO_3_ and Bi_1.2_Fe_0.98_Mn_0.02_O_3_ targets were used. In previous characterization of BFMO films prepared using the same combinatorial sputtering approach, the incorporated Mn content was estimated to be approximately 1.2–1.3% across the wafer.^12^ Deposition was performed at a substrate temperature of 500 °C with an RF power of 30 W applied to each target. The Ar/O_2_ gas flow ratio and working pressure were set to 20:1 and 1 Pa, respectively. Finally, the Pt top electrode was deposited by sputtering and patterned via Ar dry etching. The BFMO thickness was selected to remain relevant to the MEMS-oriented BFO/BFMO film platform, in which submicrometer- to micrometer-scale piezoelectric films are typically required for sufficient electromechanical response.

### 2.2 Material Characterization

The crystalline structure of the films was investigated using X-ray diffraction (XRD) with 2θ-ω scan and φ-scan measurements (X’Pert-MRD, Philips, Almelo, Netherlands), using Cu–K_α1_ radiation. The surface morphology and cross-sectional microstructure were characterized by field-emission scanning electron microscopy (FE-SEM, Zeiss Merlin and Gemini 500, Jena, Germany).

Highly accelerated lifetime testing (HALT) was conducted to study the lifetimes and degradation mechanisms of the BFMO film. Testing was performed using an aixHALT system (aixACCT Systems GmbH, Aachen, Germany). The deposited wafer was cleaved into small pieces, with each piece containing 13 patterned electrodes. The pieces were attached to a dual-in-line package (DIP) by using silver paint. Gold wire bonding was used to connect individual top electrodes to the DIP. The leakage currents were recorded under DC fields of 100 kV/cm at temperatures of 333, 343, 353, or 363 K. Weibull analysis was used to calculate the median time to failure (MTTF), where device failure was defined as the time the leakage current increased by 2 orders of magnitude with respect to the minimum leakage current.

To investigate the degradation mechanism, the activation energy was extracted from HALT data under a field-up bias. The activation energy for electrical degradation was determined using the empirical relationship suggested by Prokopowicz–Vaskas.^44^

1 $$\frac{t_{1}}{t_{2}}={\left(\frac{V_{1}}{V_{2}}\right)}^{n}\exp\left[\frac{E_{a}}{k_{B}}\left(\frac{1}{T_{2}}-\frac{1}{T_{1}}\right)\right]$$

where *t*_1_, *t*_2_ are the times to failure at voltage *V*_1_, *V*_2_, n is the voltage acceleration exponent, and *E*_*a*_ is the activation energy. When the voltage is constant, this equation can be simplified to an Arrhenius-type equation for the median time to failure (MTTF):

2 $$\frac{1}{\mathrm{MTTF}}=K_{0}\exp\left[-\frac{E_{a}}{k_{B}T}\right]$$

where *K*_0_ is the degradation rate constant. Thermally stimulated depolarization current (TSDC) measurements were carried out using an electrometer (B2985B, Keysight Technologies, Santa Rosa, CA, USA) to investigate the origin and spatial distribution of depolarization processes in the BFMO films. To elucidate the mechanisms underlying the observed TSDC peaks, such as trapped charges, defect dipoles, or space charge, the electric field dependence of the TSDC response was systematically investigated. Samples were degraded under 25.0, 37.5, or 50.0 kV/cm at 353 K for 15 min and then cooled to room temperature with the electric field maintained. The electric field was subsequently removed, and the samples were short-circuited. The temperature corresponding to the maximum TSDC current (*T*_max_), was monitored as a function of the poling field. The TSDC process can be described using the following equations.^45^

3 $$J\left(T\right)=sn_{0\;}\exp\left(\frac{-E_{a}}{k_{b}T}\right)\exp\left[-\frac{s}{\beta }\int_{T_{0}}^{T_{1}}\left(-\frac{E_{a}}{k_{b}T}dT\right)\right]$$

4 $$s=\beta \frac{E_{a}}{k_{b}T_{max}^{2}\exp\left(\frac{E_{a}}{k_{b}T_{max}}\right)}$$

where β is the heating rate, *n*_0_ is the concentration of dipoles, and *s* is a geometrical factor depending on the dipole orientation. In this equation, the initial rise of the current with temperature is governed by the first exponential term, allowing the activation energy to be estimated from the slope of a plot of ln *J* and 1/*T* (known as the “initial rise method”). Furthermore, the full width at half-maximum (FWHM) method was used to determine the activation energy of the defect peak.^46^ A background correction was applied to eliminate the pyroelectric response from the material; the corrected peak was then fitted using a Gaussian model.

When *T*_max_ shifts to high temperature with increasing poling field, the depolarization current corresponds to space charge; when *T*_max_ remains constant with an increasing poling field, the peak is attributed to the depolarization of defect dipoles; and when *T*_max_ decreases with an increasing poling field, the peak is due to trapped charges.^47^ Finally, the depolarization current was measured while heating at a constant rate up to 573 K. The measurements were repeated for opposite polarization directions to probe the local defect chemistry near the top and bottom electrodes.

The DC leakage current was measured using a picoammeter (Model 4140B, Hewlett–Packard, Palo Alto, CA, USA) at different temperatures (303 to 343 K) to investigate the conduction mechanism. For each measurement, the applied voltage was held for 60 s to allow the transient polarization currents to decay, ensuring that the measured current represented the steady-state leakage current. The voltage was then incrementally increased until the dielectric breakdown of the film occurred. The measurement was repeated for different polarities to understand the difference in conductivity at the two interfaces.

The conduction mechanism for the field-up bias, with the top electrode grounded and a positive voltage applied to the bottom electrode (and vice versa for the field-down bias), was assessed by fitting the *J*–*E* relationship to a Poole–Frenkel model.^48^

5 $$\ln\left(\frac{J}{E}\right)=\ln C_{T}-\frac{qf_{B}^{P-F}}{k_{B}T}+\frac{q\sqrt{\frac{qE}{\varepsilon_{0}\varepsilon_{\infty }\pi }}}{k_{B}T}$$

where *C_T_* is a constant and $f_{B}^{P-F}$ is the depth of the trap. The validity of the Poole–Frenkel model was evaluated by extracting the optical dielectric constant, *ε*_∞_ and comparing the corresponding refractive index, $n=\sqrt{\varepsilon_{\infty }}$ , with the reported literature values for BFMO, ranging from 2.6 to 3.2.^49^

Modulus spectroscopy was employed to gain deeper insight into the prevailing bulk charge transport mechanisms in the BFMO film. This technique is particularly effective for highlighting bulk relaxation processes.^50^ Modulus spectroscopy measurements were performed using a ModuLab XM (Ametek, Berwyn, PA, USA) over a wide frequency range (0.1 Hz to 1 MHz) at temperatures from 303 to 503 K. At each temperature, the frequency-dependent complex modulus was recorded to identify relaxation processes associated with defect-mediated charge transport in the films. The characteristic relaxation frequency *f_r_* which represents the peak position of M″ is given by,

6 $$f_{r}=\frac{1}{2\pi RC}=\frac{\sigma }{2\pi \varepsilon_{0}\varepsilon_{r}}$$

where *R* and *C* are resistance and capacitance, *σ* is the conductivity, and *ε_r_* is the relative permittivity at the measured temperature. The activation energy associated with the relaxation process was extracted from Arrhenius fitting of the temperature-dependent conductivity.

Charge-based deep-level transient spectroscopy (Q-DLTS) was conducted to probe trap states in the BFMO film. In this measurement, the applied voltage pulse fills accessible trap states in the BFMO film and/or near the BFMO/electrode interfaces. After the pulse is removed, trapped carriers are thermally emitted, producing a charge-decay transient. Therefore, the Q-DLTS signal in the present structure is interpreted as arising from trap states accessible within the ferroelectric capacitor geometry.^51–53^ Details of the procedure are described elsewhere.^51–53^ For the measurement, a charging voltage of 8 V was applied for 300 ms, and then the transient discharge was recorded across a temperature range of 150 to 325 K. The experiment was repeated for a higher temperature range (300 to 900 K), under similar charging voltage and pulsing time, to probe deeper levels. The relaxation of transient charges was converted to a DLTS signal (Δ*Q*), with specific rate windows at each temperature as follows:

7 $$\Delta Q=Q\left(t_{1}\right)-Q\left(t_{2}\right)$$

where the rate window corresponds to the time interval between *t*_1_ and *t*_2_. Q-DLTS spectra were acquired for various rate window settings, and the resulting signals were analyzed by fitting the peaks with a Gaussian distribution to determine their energy levels. The characteristic emission time constant, *τ_m_*, for traps was estimated using the following relationships:^51^

8 $$\tau_{m}=\frac{1}{\sigma T^{2}N_{c}V_{th}}\exp\left(\frac{\Delta E}{kT}\right)$$

9 $$\tau_{m}=\frac{t_{2}-t_{1}}{\ln\left(t_{2}/t_{1}\right)}$$

where *V_th_* is thermal velocity, *N_c_* is the density of states, and Δ*E* is the trap activation energy. A pronounced Q-DLTS peak was observed for the rate window with a constant ratio of *t*_2_/*t*_1_ = 10 (see Figure 7). The peak temperatures for different rate windows were analyzed using an Arrhenius plot, enabling the extraction of the trap activation energy from the slope.

## 3 Results and Discussion

Figure 2 a, b shows the XRD 2θ-ω and φ-scan profiles of the BFMO film. The 2θ-ω scan confirms the growth of a strongly textured (100) BFMO film without any secondary phases or misoriented grains. The φ-scan results show four 202 diffraction peaks aligned from the Si substrate through the TiN/Pt/LNO/BFMO multilayer stack, indicating cube-on-cube epitaxial growth. This epitaxial relationship indicates that the present BFMO film is different from conventional randomly oriented polycrystalline sputtered films. In randomly oriented polycrystalline films, grain boundaries can strongly affect leakage current by providing conductive paths or by blocking oxygen-vacancy motion. Such effects are especially important when grain-boundary networks interrupt the current path between the top and bottom electrodes. Therefore, the leakage and degradation analyses in this BFMO epitaxial film will focus mainly on point defects, oxygen-vacancy redistribution, and asymmetric electrode/interface effects rather than grain-boundary-controlled leakage.

**Figure 2:**
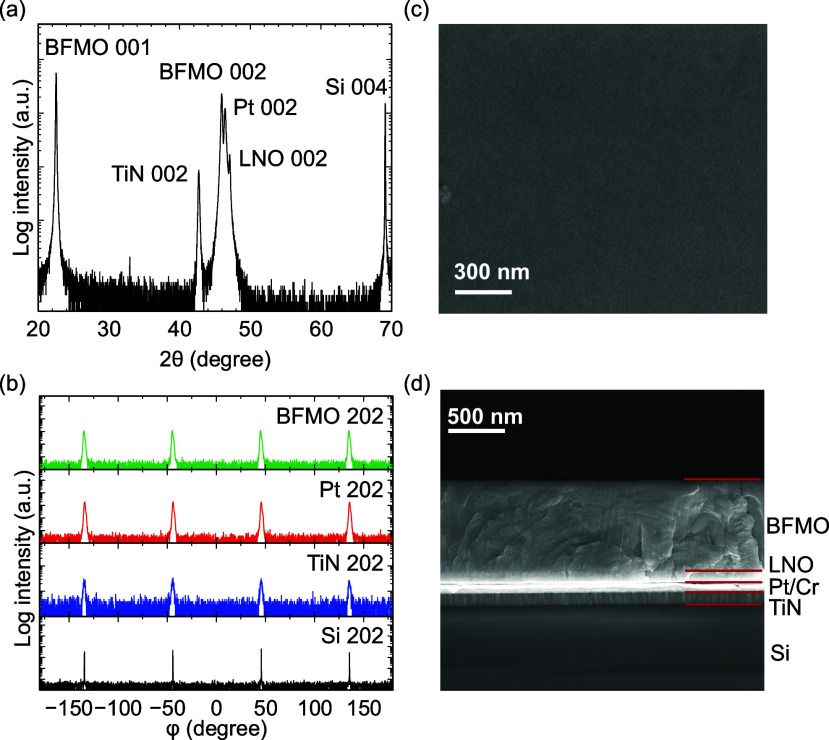
(a) 2θ-ω and (b) φ-scan profile of BFMO film, scanning electron microscopy (SEM) image of (c) top surface and (d) cross-sectional of the BFMO film.

The surface morphology examined by SEM (Figure 2c) shows that the film is dense and free of visible cracks or pores. Furthermore, atomic force microscopy (AFM) characterization shows that the film exhibits an RMS roughness of 2.5 nm, confirming a smooth surface morphology (as shown in Figure S1). The cross-sectional SEM images (Figure 2d) show the thickness of each layer; the TiN, CrV, Pt, LNO, and BFMO layers are 109, 25, 78, 94, and 809 nm, respectively. This BFMO thickness is representative of the relatively thick film geometry used in BFO/BFMO piezoelectric MEMS. Therefore, the present film should not be interpreted in the same manner as an ultrathin, fully coherent BFO film, in which an unrelaxed epitaxial lattice mismatch strain dominates. Instead, any residual stress in this Si-based film stack is expected to be governed primarily by thermal expansion mismatch during cooling, resulting in tensile residual strain, as discussed in detail for previous BFO/BFMO.^12^ In the previous work, Mn incorporation was shown to modify the structural stability of BFO on Si, including the stabilization of the *R*3*c* phase and the modification of the strain-induced *R*3*c*-to-M_B_ transition behavior. Furthermore, the cross-sectional image reveals a columnar microstructure consistent with highly oriented out-of-plane growth. These structural and morphological results demonstrate the successful fabrication of epitaxial BFMO films suitable for subsequent electrical reliability studies.

The BFMO film exhibited a relatively low dielectric constant of 103, with a dielectric loss tangent of 0.012. The ferroelectric properties were confirmed by well-shaped polarization-electric (P-E) hysteresis loops, with 2*E_c_* = 179 kV/cm and 2*P_r_* = 84 μC/cm^2^ (see Figure S2). Table S2 summarizes representative values of coercive field, remanent polarization, dielectric constant, and dielectric loss reported for BFO and Mn-doped BFO thin films.^30,54–60^ Compared with the representative Mn-doped BFO thin films listed in Table S2, the present film exhibits a relatively low dielectric constant and dielectric loss, a comparatively low coercive field, and a moderate remanent polarization.

The long-term reliability results obtained from the HALT measurements indicate a significant dependence of the DC degradation lifetime on the electric field polarity, as shown in Figure 3a. Notably, the leakage current was substantially lower for the field-down direction when compared to the field-up bias, with no appreciable signs of resistance degradation under identical degradation conditions, even after extended stress. This pronounced asymmetry in the DC degradation could arise from the nonuniform distribution of defects and oxygen vacancies in the film, which ultimately governs the failure mechanism of the films. For the field-up bias, the measured MTTFs of the BFMO films are 949, 455, 328, and 191 s at temperatures of 333, 343, 353, and 363 K, respectively. The corresponding Weibull plots illustrate the statistical distribution of failure events (Figure 3b). An Arrhenius analysis of the MTTF data (Figure 3c) yields an activation energy for degradation under field-up bias of *E*_a_ = 0.54 ± 0.01 eV, consistent with reported activation energies for oxygen-vacancy migration in BFO thin films.^61^ These results suggest that the observed polarity dependence arises from an asymmetric distribution of oxygen vacancies.

**Figure 3:**
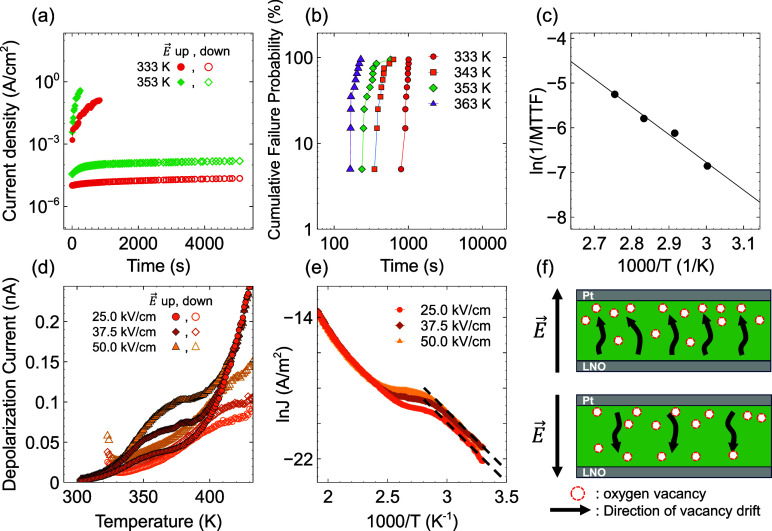
Degradation and defect spectroscopy under DC stress. (a) leakage current density versus time during DC degradation, (b) Weibull cumulative failure probability as a function of time, (c) Arrhenius plot of the MTTF, (d) TSDC at 353 K for 15 min under fields of 25.0, 37.5, 50.0 kV/cm, (e) initial-rise analysis used to estimate the activation energy from the TSDC data, (f) schematic illustrating the asymmetric oxygen-vacancy distribution and polarity-dependent migration (field-up vs field-down).

To identify the mobile species responsible for the observed polarity-dependent degradation, thermally stimulated depolarization current (TSDC) measurements were performed. After electrical degradation under a field-up electric field of 25–50 kV/cm at 350 K for 15 min, the TSDC data exhibit a single depolarization-current peak centered near 360 K. To elucidate its physical origin, the dependence of the peak temperature (*T*_max_) on the applied degradation field was examined. *T*_max_ shifts systematically to higher temperatures with increasing poling electric field, consistent with a space-charge depolarization process driven by ionic transport, most plausibly oxygen-vacancy migration.

The activation energy associated with the TSDC peak was extracted using both the initial-rise and full-width-at-half-maximum (FWHM) methods. The two approaches yield similar values: the initial-rise analysis gives 0.58 ± 0.01 eV (Figure 3e), whereas the FWHM method yields 0.54 ± 0.01 eV (Figure S3). These values are consistent with the activation energy derived from the HALT measurements, providing strong evidence that electrical degradation and eventual failure are governed by oxygen-vacancy migration.

Furthermore, the TSDC spectra for the field-down bias revealed a similar right-shifting peak with an intensity approximately half that of the field-up direction. Since the area under the curve is proportional to the concentration of mobile oxygen vacancies, the lower-intensity peak signifies a lower oxygen vacancy concentration near the bottom electrode. As illustrated in Figure 3f, the higher vacancy concentration near the top electrode enhances ionic migration and electron trapping under the field-up bias, thereby accelerating resistance degradation and shortening the lifetime. When the polarity is reversed, regions with a lower vacancy concentration govern the electrical response, resulting in greater resistance stability and a higher lifetime.

The diffusion coefficient of oxygen vacancies was calculated using the Nernst–Einstein equation as follows:^46^

10 $$D_{V}=\frac{kT}{2e}u_{v}=\frac{kTL}{2e\tau E},\;u_{v}=\frac{v}{E}$$

where *k* is the Boltzmann constant, *v* and *u_v_* are the velocity and mobility of mobile ions, *e* is the elementary charge, *T* is the temperature, and *E* is the electric field. By assuming that velocity is given by *L*/*τ* and the oxygen vacancy migrated across the sample thickness *L* = 809 nm, in the degradation time of *τ* = 15 min, at 353 K, the diffusion coefficient of the oxygen vacancies was calculated to be 2.7 × 10^–14^ cm^2^/s, which is significantly higher than those reported for PZT ceramic (1.1 × 10^–18^ cm^2^/s)^62^ at the same temperature and also exceeds the values reported for PZT-based films (8–9 × 10^–15^ cm^2^/s) which were obtained at higher temperatures of 480–510 K.^45,52,53,63,64^ Moreover, the TSDC peak in the BFMO film emerges under relatively mild degradation conditions when compared to PZT-based films, which typically require more severe electrical or thermal stress to reveal a relaxation peak. Hence, the long-term reliability of the BFMO is governed by enhanced mobility and rapid diffusion of oxygen vacancies.

To determine whether the same asymmetric defect distribution also governs steady-state charge transport, the conduction mechanisms were analyzed under opposite electric field polarities. As shown in Figure 4a, the nonuniform distribution of oxygen vacancies is reflected in the DC leakage-current behavior of the BFMO film, which shows a pronounced polarity dependence with a substantially higher current density in the field-up direction. To evaluate the effect of Mn incorporation, the J–E characteristics of a pristine BFO control film prepared using the same combinatorial sputtering approach were also measured. As shown in Figure S4, the BFMO film exhibits a lower leakage current than the pristine BFO control under comparable measurement conditions. This comparison supports the interpretation that Mn incorporation reduces leakage in the present BFO film. Furthermore, to evaluate the bias-history dependence of this response, forward and reverse voltage-sweep J–E characteristics were measured and are shown in Figure S5. The sweep data show a small hysteresis; however, the overall polarity-dependent leakage behavior remains unchanged. In addition, the steady-state J–E characteristics were compared before and after 10^9^ unipolar triangle wave voltage cycles at 100 kHz, 25 kV/cm, and room temperature (Figure S6). The leakage current remains on the same order of magnitude, and no catastrophic leakage increase or breakdown is observed after cycling. These results indicate that the BFMO film maintains stable leakage-current behavior under the tested cycling conditions, supporting the use of the steady-state J–E data in Figure 4 for conduction-mechanism analysis.

**Figure 4:**
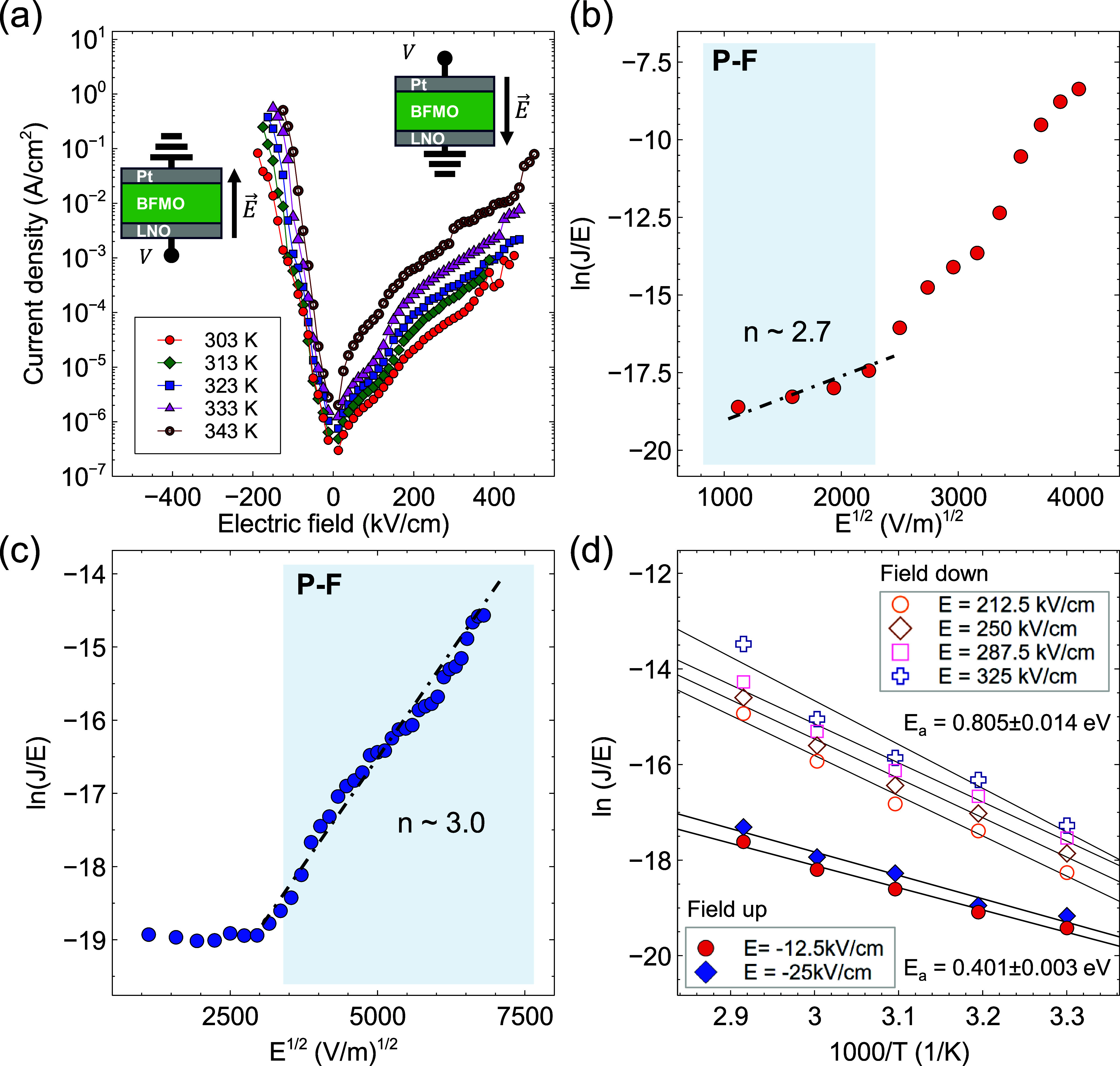
Polarity-dependent leakage-current behavior and Poole–Frenkel analysis of the BFMO film. (a) Current density as a function of the electric field under field-up and field-down bias. Poole–Frenkel fitting results for (b) field-up and (c) field-down bias over the corresponding electric field ranges. (d) Arrhenius analysis of the Poole–Frenkel trap ionization energy for field-up and field-down; each fitted line corresponds to one bias polarity.

Conduction mechanism analysis identifies Poole–Frenkel emission as the dominant charge transport mechanism for *E* < 50 kV/cm under field-up and E > 100 kV/cm under field-down bias (Figure 4b, c). The extracted trap ionization energy is 0.40 ± 0.01 eV for the field-up bias, which aligns with the reported values for the oxygen vacancy trap state (Figure 1).^28^ For the field-down polarity, the trap ionization energy is 0.81 ± 0.01 eV, which is associated with electron excitation from the Fe trap state (Figure 4d). The difference between the trap ionization energies for the Poole–Frenkel conduction observed for the two polarities is a consequence of the asymmetric oxygen vacancy distribution. The relatively oxygen-vacancy-depleted region near the bottom electrode suppresses oxygen-vacancy-mediated transport, thereby shifting conduction to the next accessible trap state, which, according to the energy band diagram, is the ${\mathrm{Fe’}}_{\mathrm{Fe}}$ levels. In contrast, the region near the top electrode supports conduction predominantly through abundant, low-energy oxygen-vacancy-related trap states. Such a nonuniform distribution may be introduced during film growth. The LNO bottom electrode is an oxide electrode and may act as an oxygen-exchange layer or oxygen reservoir during processing, whereas the Pt top electrode is deposited after BFMO growth and does not provide an equivalent oxygen reservoir. Therefore, it is plausible that the region near the Pt/BFMO interface is relatively oxygen-vacancy-rich, while the region near the BFMO/LNO interface is comparatively oxygen-vacancy-poor. This nonuniform oxygen-vacancy distribution may also contribute to the imprint observed in the P–E hysteresis loop, which is shifted by 20.5 kV/cm, as shown in Figure S2. However, the imprint and initial electrical asymmetry may also arise from other coupled effects, including defect dipoles, space-charge accumulation, electrode work-function differences, and growth-induced built-in fields.^64^

Furthermore, as shown in Figure 5, the activation energy calculated from modulus spectroscopy (0.80 ± 0.01 eV) indicates that conduction through the bulk of the film is controlled by electron emission from the ${\mathrm{Fe’}}_{\mathrm{Fe}}$ trap states, consistent with the Poole–Frenkel activation energy observed in the J-E analysis. However, because the carrier mobility, ionization fraction, defect charge state, and relative electronic/ionic contributions are not independently known, the modulus spectra are not used here to calculate an absolute defect concentration.

**Figure 5:**
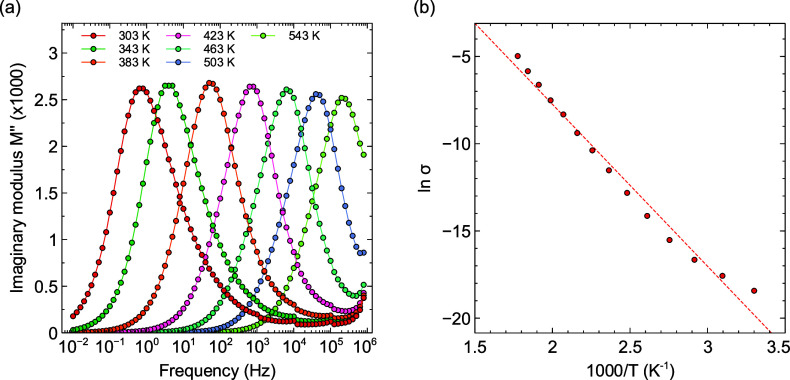
(a) Temperature dependence of imaginary modulus as a function of frequency, (b) Arrhenius plots for conductivity of BFMO film.

The conduction for the field-up direction for *E* > 50 kV/cm was consistent with the space charge limited conductivity (SCLC) mechanism as shown in Figure 6a. This was verified by observing the temperature-dependent shift of the trap-filled limiting voltage (*V*_TFL_), a characteristic signature of trap-controlled SCLC.^65^ The saturation of shallow traps for the field-up bias drives a transition from Poole–Frenkel at intermediate fields to space charge limited conduction at higher fields. With increasing temperature, the free carrier density in the film rises, promoting progressive trap filling and leading to a systematic reduction in the *V_TFL_* (Figure 6b). The activation energy calculated from the plot of ln *J* vs 1/*T* was about 0.97 ± 0.11 eV (Figure 6c), which is in the range of ${\mathrm{Fe}}_{\mathrm{Fe}}^{\prime }$ trap states. These results indicate that once shallow traps are filled, carrier transport becomes limited by deeper trap states, such as ${\mathrm{Fe}}_{\mathrm{Fe}}^{\prime }$ sites, leading to SCLC at elevated fields.

**Figure 6:**
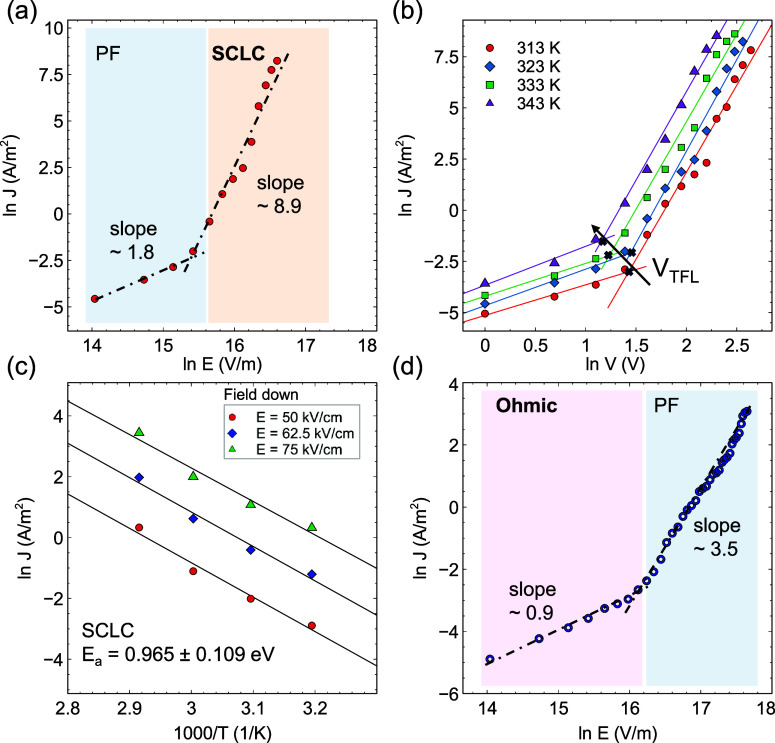
(a) Fitting results for space charge limited current (SCLC), (b) current density and electric field dependence of temperature used to extract *V_TFL_*, (c) estimation of activation energy corresponding to the SCLC mechanism, and (d) fitting results for Ohmic conduction in field-down bias at E < 100 kV/cm.

For field-down bias, the leakage current follows Ohmic behavior for *E* < 100 kV/cm, as evidenced by the proportional scaling of ln *J* with ln *E* (Figure 6d). This behavior suggests that charge carriers are transported as free electrons in the conduction band with minimal involvement of trap states. This is consistent with the low concentration of oxygen vacancies near the bottom electrode, which reduces the availability of shallow traps that would otherwise facilitate trap-assisted conduction.

Moreover, Q-DLTS revealed the presence of additional traps within the film, and a shallow trap level is detected from the peak around 180 K as shown in Figure 7a; this corresponds to an activation energy of 0.17 ± 0.03 eV, which is associated with the $V_{Bi}^{’’’}$ defect level. The presence of $V_{Bi}^{’’’}$ is plausibly linked to Bi_2_O_3_ volatilization during film processing, promoting the concurrent formation of $V_{Bi}^{’’’}$ and $V_{O}^{\cdot \cdot }$ . High-temperature Q-DLTS measurements further reveal a deep trap level with an activation energy of 1.55 ± 0.12 eV, assigned to the ${\mathrm{Mn}}_{\mathrm{Fe}}^{\times }$ site. Such deep traps are difficult to probe due to a higher threshold energy for the trapped charges to overcome to reach the conduction band; however, with increasing temperature and a shift in the Fermi level, the probability of electron emission from the ${\mathrm{Mn}}_{\mathrm{Fe}}^{\times }$ site increases, enabling these trap states to be detected.

**Figure 7:**
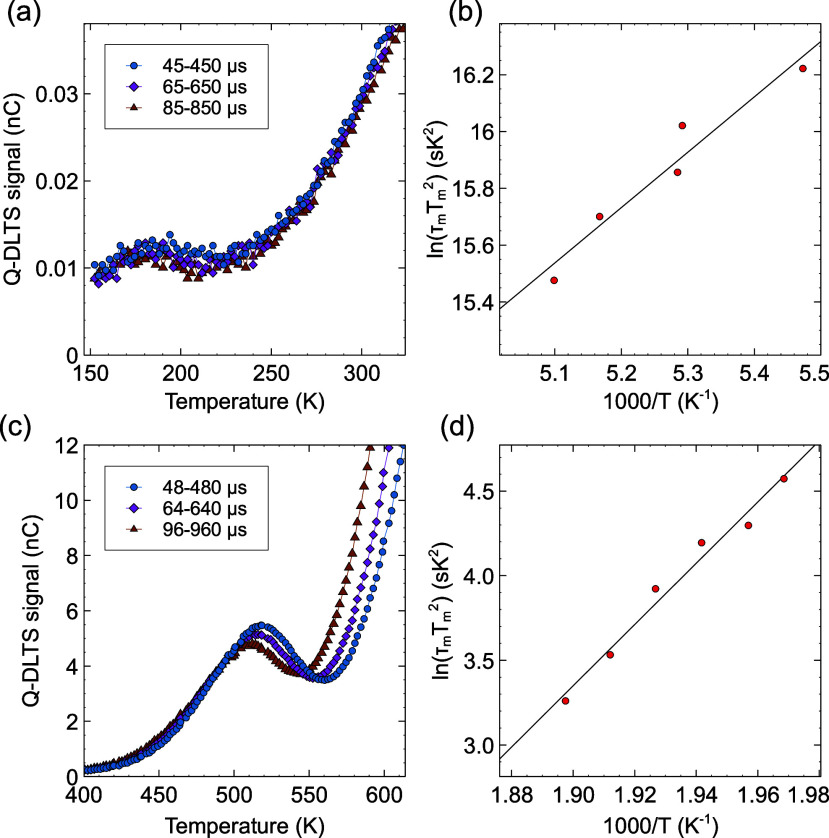
(a) Charge-based deep level transient spectroscopy (Q-DLTS) at temperatures from 150 to 325 K under various rate windows, (b) Arrhenius plot of the Q-DLTS signal. (c) Charge-based deep level transient spectroscopy (Q-DLTS) at temperatures from 400 to 650 K under various rate windows, and (d) Arrhenius plot of the Q-DLTS signal.

As can be seen from the results, the conductivity behavior of BFMO is influenced by multiple electrically active defect states, including oxygen-vacancy-related and Fe-related states. The Q-DLTS results are consistent with the presence of a deep Mn-related trap level, which may localize electrons and reduce the contribution of Fe^2+^/Fe^3+^ hopping to leakage conduction. This interpretation is consistent with prior X-ray photoelectron spectroscopy (XPS) studies of Mn-doped BFO,^41,66^ which reported mixed Mn valence states (Mn^2+^, Mn^3+^, and Mn^4+^) and a reduction in Fe^2+^-related defect contributions at low Mn substitution levels. In addition, the oxygen-vacancy diffusion coefficient extracted in this study (2.7 × 10^–14^ cm^2^/s) is an order of magnitude lower than values previously reported for Ca-doped BFO films (2 × 10^–13^ cm^2^/s) at the same temperature of 353 K.^67^ This suggests that Mn may participate in defect association with oxygen vacancies and thereby reduce oxygen-vacancy mobility. However, because of the low Mn concentration in the present BFMO film, quantitative determination of the Mn valence-state distribution by XPS or related spectroscopic techniques would be highly uncertain. Thus, the Mn-related assignment discussed here is based on the electrical response, Q-DLTS activation energy, and defect-chemistry considerations, rather than on a direct spectroscopic determination of a specific Mn oxidation state. In comparison with prior reports on the reliability under DC fields of PZT films,^52,53,62,68^ the operational lifetime of BFMO is considerably shorter under comparable stress conditions. This suggests that BFMO is better suited for applications that avoid sustained high-field operations, such as sensors and energy harvesters, rather than actuators. This allows the comparatively low relative permittivity of the BFMO film, relative to PZT, to be employed to increase the voltage sensitivity of sensors and should also improve its figure of merit for energy harvesting, while avoiding the need to regularly apply high electric fields, as is required in using the converse piezoelectric effect for actuators.

## 4 Conclusions

In this work, the conduction mechanisms and DC resistance degradation of epitaxial BFMO films were systematically investigated with an emphasis on their reliability for piezoelectric MEMS applications. Multiple characterization techniques, including temperature-dependent leakage current measurements, TSDC, Q-DLTS, modulus spectroscopy, and HALT, were employed to elucidate the links between defect chemistry, charge transport, and electrical degradation in BFMO films.

A nonuniform oxygen-vacancy distribution across the film thickness was identified as the underlying origin of polarity-dependent charge transport, asymmetric leakage current, and reliability in BFMO. For field-up bias, Poole–Frenkel emission is governed by electron trapping at abundant oxygen-vacancy–related shallow trap levels. At higher electric fields, progressive filling of these shallow traps drives a transition from Poole–Frenkel emission to trap-controlled space-charge-limited conduction, in which transport becomes limited by deeper ${\mathrm{Fe}}_{\mathrm{Fe}}^{\prime }$ states. For field-down bias, in contrast, the reduced oxygen-vacancy concentration near the bottom electrode suppresses vacancy-mediated transport; Poole–Frenkel emission therefore proceeds predominantly through ${\mathrm{Fe}}_{\mathrm{Fe}}^{\prime }$ trap levels over a broad field range, following an Ohmic low-field regime. In addition, Q-DLTS resolved further defect levels, including shallow $V_{Bi}^{’’’}$ and deep ${\mathrm{Mn}}_{\mathrm{Fe}}^{\prime }$ traps, that contribute to bulk conductivity.

The same nonuniform oxygen-vacancy profile also governs the polarity dependence of resistance degradation and lifetime. Owing to the initially $V_{O}^{¨}$ -rich region near the top electrode, field-up bias promotes rapid $V_{O}^{¨}$ drift and redistribution, thereby accelerating degradation kinetics and yielding a shorter lifetime. In contrast, under field-down bias, oxygen vacancies must first migrate and accumulate at the bottom interface before comparable degradation occurs, resulting in slower degradation and a longer lifetime.

Overall, these findings provide material-level reliability insights for BFMO films being considered for piezoMEMS actuator applications. Although the present work does not introduce a new MEMS device demonstration, it identifies defect-mediated charge transport and polarity-dependent degradation mechanisms that are directly relevant to the stable operation of BFO/BFMO-based piezoMEMS devices under applied electric fields. The results therefore provide guidance for future optimization of defect chemistry, electrode/interface design, and operating bias conditions in BFMO-based piezoMEMS platforms. In particular, controlling oxygen vacancy concentration and migration pathways will be essential for extending BFO/BFMO films from passive sensing and energy-harvesting applications toward more actively driven MEMS devices, such as actuators and ultrasonic transducers.

## Supplementary Material



## Notes

Representative piezoelectric properties of BFO-based films; AFM surface topography; dielectric and ferroelectric characterization; comparison of BFO-based films; TSDC background subtraction and activation-energy extraction; pristine BFO/BFMO steady-state J–E comparison; forward/reverse voltage-sweep J–E characteristics; leakage-current stability after 10^9^ unipolar electrical cycles (PDF)

## #

Department of Electrical and Electronic Information Engineering, Toyohashi University of Technology, Toyohashi, Aichi 441-8580, Japan
