# Supplementary material for: Polarity-Dependent Charge Transport and Resistance Degradation Enabled by Oxygen-Vacancy Gradients in BiFeO3 Films
Source: ACS Appl Mater Interfaces. 2026 Jun 23;18(26):36891–901. doi: 10.1021/acsami.6c00718 (PMC13352513; doi:10.1021/acsami.6c00718)
Supplement: Supplementary file 1 [file am6c00718_si_001.pdf]

Supporting information

Polarity-Dependent Charge Transport and Resistance Degradation Enabled by Oxygen-Vacancy Gradients in $BiFeO_3$ Films

Sengsavang Aphayvong,[†] *Abhyuday Verma,*[‡] *Kae Nakamura,*[‡] *Ali Habib Akhyari,*[‡] *Takeshi Yoshimura,*[†,+] *Susan Trolier-McKinstry*[‡] *and Betul Akkopru-Akgun*[*,‡]

[†] Department of Physics and Electronics Engineering, Osaka Metropolitan University, Sakai-shi, Osaka 599-8531, Japan

[‡] Department of Materials Science and Engineering, The Pennsylvania State University, University Park, Pennsylvania 16802 USA

[+]Present address:
Department of Electrical and Electronic Information Engineering, Toyohashi University of Technology, Toyohashi, Aichi 441-8580, Japan

*Corresponding Author. Email: bua134@psu.edu

Table S1. Representative piezoelectric properties of BFO-based films reported in the literature.

Reference	Material / composition	$BiFeO_3$ thickness (nm)	$\|e_{31,f}\|$ (C/m^2)
Ujimoto et al.[13]	(100) $BiFeO_3$ epitaxial film on $SrTiO_3$	500	3.5
	(111) $BiFeO_3$ epitaxial film on $SrTiO_3$	500	1.3
Yoshimura et al.[14]	Domain-engineered (100) $BiFeO_3$ film on $SrTiO_3$	350	4.0
	Domain-engineered (100) $BiFeO_3$ film on $SrTiO_3$	350	2.5
Yoshimura et al.[15]	(100)-oriented $BiFeO_3$ MEMS-pVEH on SOI	350	1.0
Aramaki et al.[16]	(100)-oriented $BiFeO_3$ film on Si	450	3.2
Yoshimura et al.[17]	(100) $BiFeO_3$ epitaxial film on $SrTiO_3$	300	4.3
Aramaki et al.[7]	(100)-oriented $BiFeO_3$ film on SOI	450	3.6
Niu et al.[6]	Stoichiometric $BiFeO_3$ film	850	2.8
Yan et al.[18]	(100)-oriented $BiFeO_3$ film on Ti foil	2200	2.2
Yan et al.[19]	(00l)-$BiFeO_3$ film on stainless steel foil	1850	1.7
	(00l)-$BiFeO_3$ film on stainless steel foil	1850	1.1
Luo et al.[20]	$BiFeO_3$ film on Si	900	2.8
**Aphayvong et al.[12]**	**Mn-doped $BiFeO_3$ epitaxial film on SOI**	**800**	**6.0**

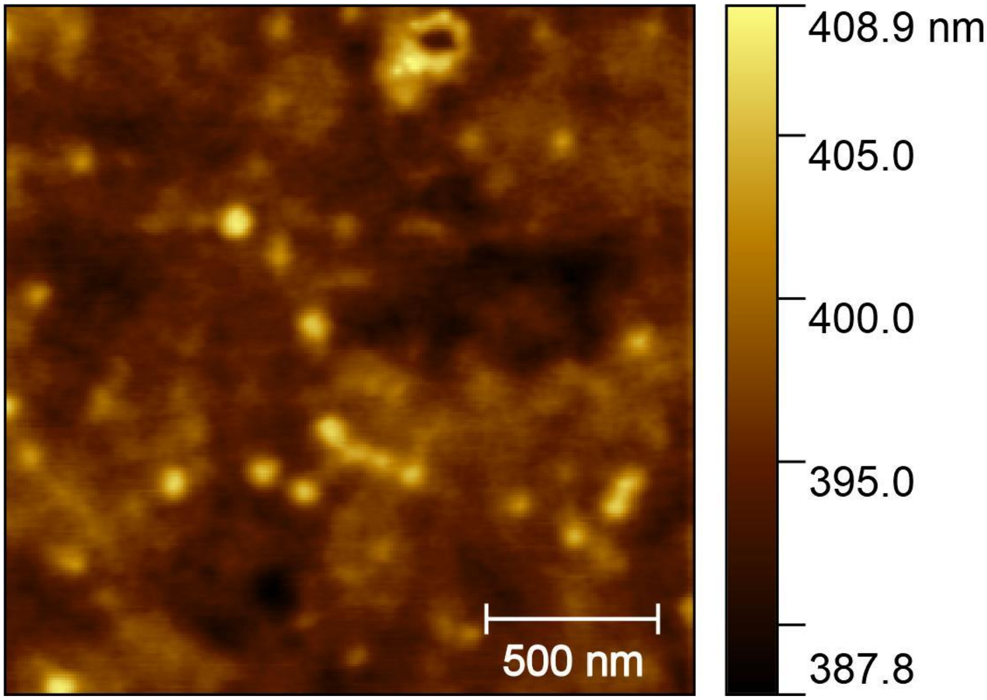

Figure S1. Atomic Force Microscopy surface topography of the BFMO film.

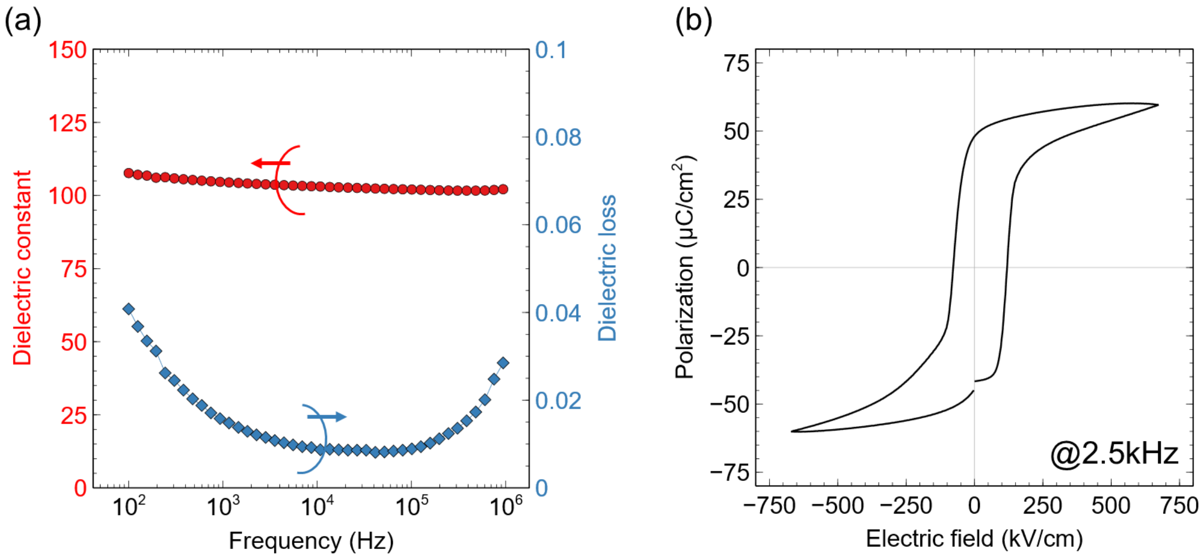

Figure S2. (a) Dielectric and (b) ferroelectric properties of the BFMO film.

Table S2. Comparison of reported BFO and Mn-doped BFO thin-film properties.

Reference	Material / Composition	Coercive field, E_c or $2E_c$	Remanent polarization, P_r or $2P_r$	Dielectric constant, ε_r	Dielectric loss, $\tan\delta$
Singh et al.[54]	$BiFeO_3$	$E_c \approx 0.4$ MV/cm	$P_r \approx 100$ μC/cm^2	$\varepsilon_r \approx 120$ on Si<br>$\varepsilon_r \approx$ 185 on sapphire	0.012
Zhang et al.[55]	$BiFeO_3$	Not reported	$2P_r = 90.9$ μC/cm^2	$\varepsilon_r = 202$	$\tan\delta = 0.057$
Lee et al.[56]	$Bi(Fe_{0.99}Mn_{0.01})O_3$	$2E_c = 630$ kV/cm	$2P_r = 139$ μC/cm^2	Not reported	Not reported
Raghavan et al.[57]	$BiFe_{0.95}Mn_{0.05}O_3$	Not reported	$2P_r = 78.37$ μC/cm^2	Not reported	Not reported
Yang et al.[58]	$BiFe_{1-x}Mn_xO_3$<br>(x = 0-0.12)	$2E_c = 134.76\text{-}196$ kV/cm	$2P_r = 9.8$ - 19.0μC/cm^2	Not reported	Not reported
Wang et al.[59]	$BiFe_{1-x}Mn_xO_3$<br>(x = 0-0.10)	Not reported	$P_r = 27$ μC/cm^2	Not reported	Not reported
Liang and Dai.[60]	$BiFe_{1-x}Mn_xO_3$<br>(x = 0-0.05)	Not reported	$2P_r = 311.5\text{-}312$ μC/cm^2	Not reported	Not reported
Nakashima et al.[30]	Mn-doped $BiFeO_3$	$2E_c = 250$ kV/cm	$2P_r = 120$ μC/cm^2	Not reported	Not reported
**This work**	Mn-doped $BiFeO_3$	$2E_c = 179$ kV/cm	$2P_r = 84$ μC/cm^2	$\varepsilon_r = 103$	$\tan\delta \approx 0.012$

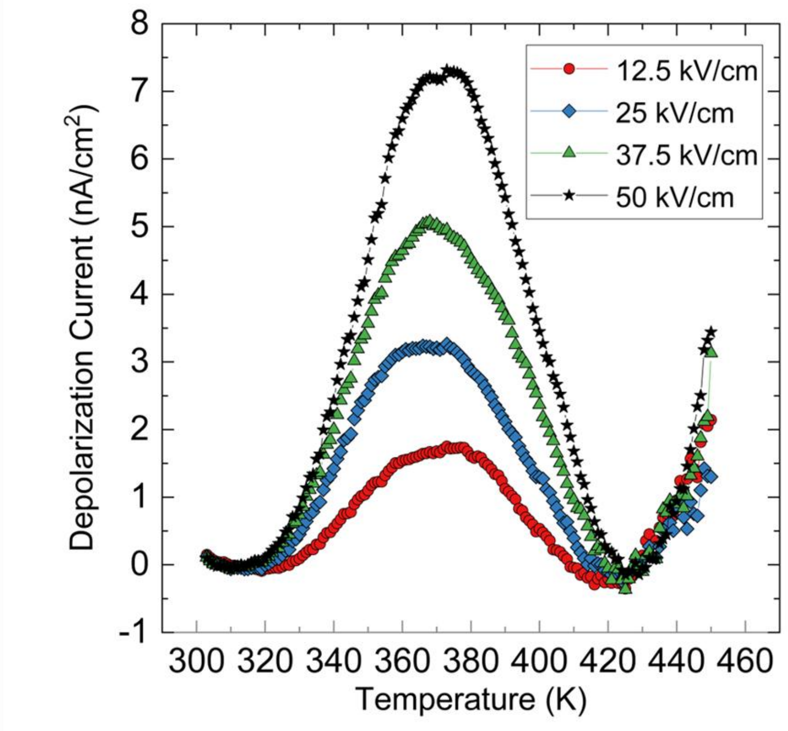

Figure S3. FWHM method for extracting activation energy from TSDC measurement after background pyroelectric subtraction.

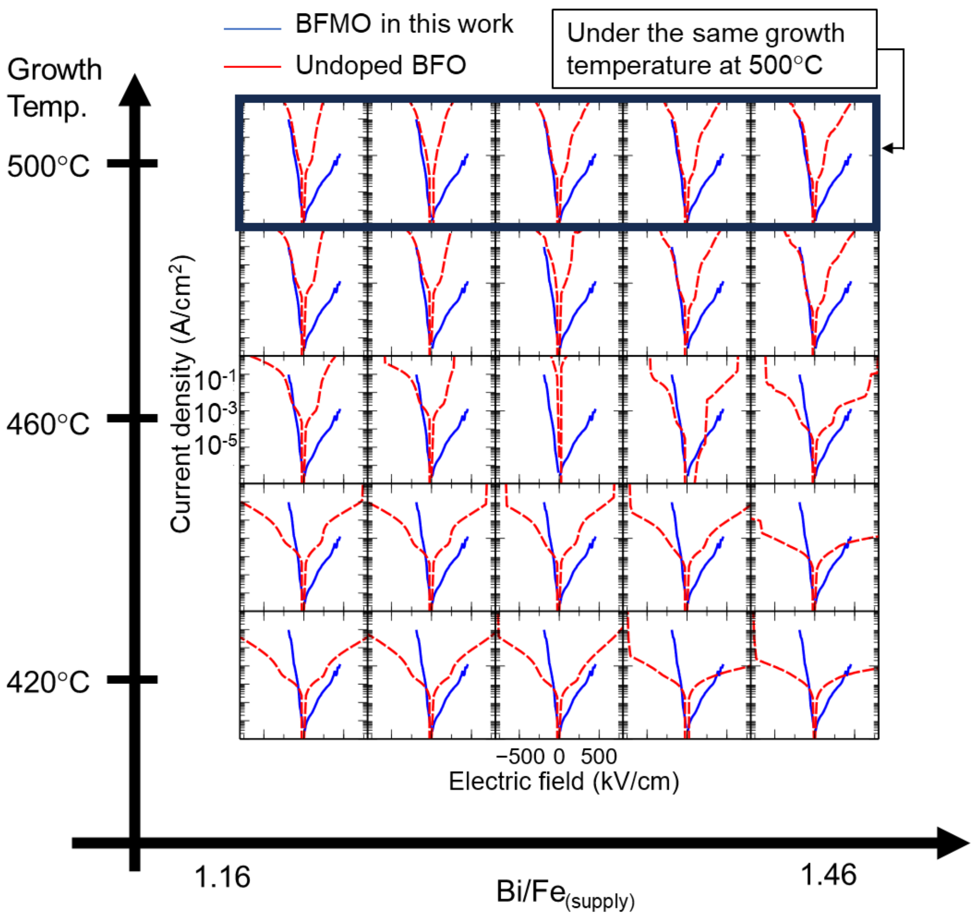

Figure S4. Comparison of steady-state J–E characteristics for pristine BFO and nominally Mn-doped BFO films prepared using the same combinatorial RF sputtering approach.

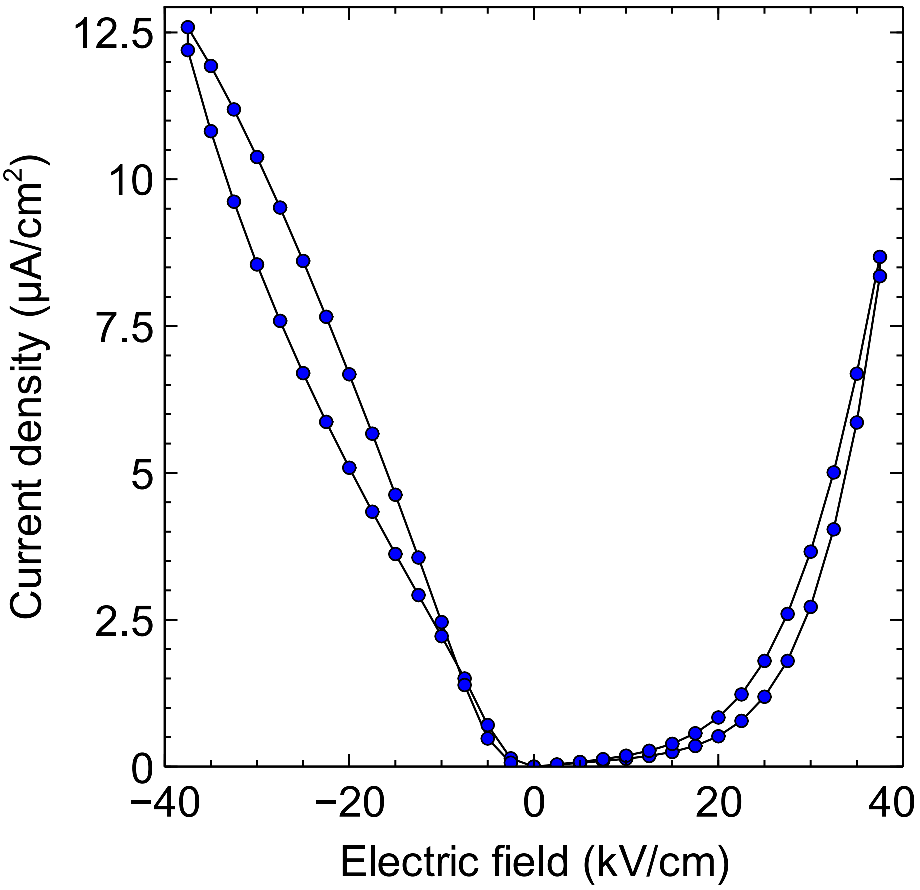

Figure S5. Forward and reverse voltage-sweep I–V characteristics of the BFMO film.

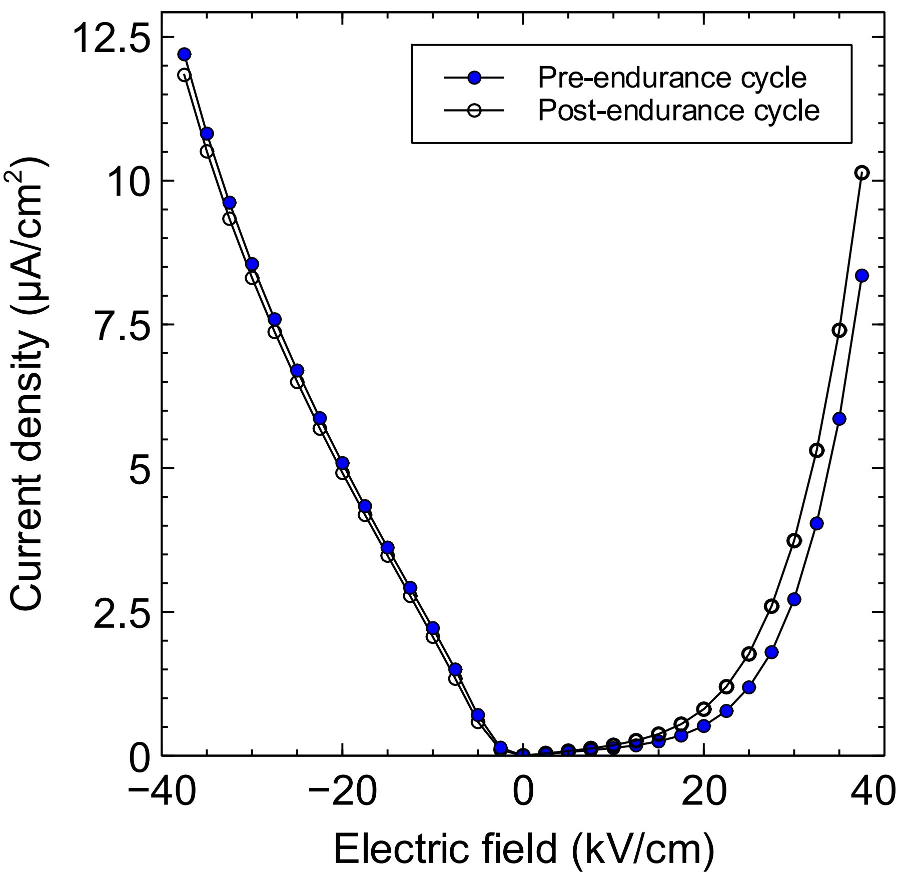

Figure S6. J–E characteristics of the BFMO capacitor measured before and after 10^9 unipolar electrical cycles.
